# Dietary habits in relation to outcome and therapy-related toxicity in patients with glioblastoma – a retrospective cohort study

**DOI:** 10.1007/s11060-025-05137-3

**Published:** 2025-07-21

**Authors:** Tareq M. Haedenkamp, Linda Götz, Tananeh Ansafi, Michael Gerken, Monika Klinkhammer-Schalke, Anna Fischl, Markus J. Riemenschneider, Julia Maurer, Martin Proescholdt, Oliver Kölbl, Nils Ole Schmidt, Ralf Linker, Elisabeth Bumes, Peter Hau

**Affiliations:** 1 Department of Neurology and Wilhelm Sander-NeuroOncology Unit, University Hospital Regensburg, Franz-Josef-Strauss-Allee 11, 93053 Regensburg, Germany; 2 Department of Neurology, Krankenhaus Barmherzige Brüder Regensburg, Regensburg, Germany; 3 Center for Quality Assurance and Health Services Research, University of Regensburg, Regensburg, Germany; 4 Department of Neuropathology, Regensburg University Hospital, Regensburg, Germany; 5 Cancer Registry, University Cancer Center Regensburg, Regensburg University Hospital, Regensburg, Germany; 6 Department of Neurosurgery, Regensburg University Hospital, Regensburg, Germany; 7 Department for Radiotherapy, Regensburg University Hospital, Regensburg, Germany

**Keywords:** Glioblastoma, Diet, Food frequency questionnaire, Therapy-related toxicity, Survival

## Abstract

**Purpose:**

Glioblastoma is the most prevalent primary malignant brain tumour in adults and bears poor survival. Data on associations of nutrition with outcome parameters are scarce. Our aim was to investigate whether dietary habits influence chemotherapy-related toxicity and overall survival in patients with glioblastoma.

**Methods:**

In this retrospective cohort study, we included glioblastoma patients who received treatment between January 2010 and December 2019. We used a 35-item food frequency questionnaire to calculate a dietary score based on Mediterranean-like diet recommendations. We analysed relationships between nutrition score, side effects and survival using chi square tests, binary logistic regression, Kaplan-Meier and multivariable Cox regression analyses.

**Results:**

From an initial population of 1,448 patients, a homogenous cohort of 128 glioblastoma cases were included in our final analysis. Patients with a higher score than the median, tending to a more Mediterranean-like diet, showed more infections (13.8% vs. 3.2%, *p* = 0.031) and a trend for more myelodepression (32.3% vs. 17.5%, *p* = 0.052). A higher score was predictive for infections (OR = 12.33, 95% CI: 1.36-111.98; *p* = 0.026). Median survival was worse in the higher score group (16.6 vs. 19.4 months, *p* = 0.004), confirmed by multivariable cox regression analysis (HR 1.60, 95% CI 1.04–2.46; *p* = 0.034).

**Conclusion:**

We identified associations between dietary patterns and chemotherapy-related toxicity as well as outcome. Our findings underscore the potential impact of nutrition on cancer treatment outcomes. Further research is needed to validate these results and to address possible underlying mechanisms.

**Supplementary Information:**

The online version contains supplementary material available at 10.1007/s11060-025-05137-3

## Introduction

Glioblastoma, IDH wild-type (CNS WHO Grade 4) (GB) represents the most common primary malignant brain tumour in adults. It is characterized by rapid growth and poor survival [1]. The incidence is between 4.5 and 7.7 per 100,000 [1]. Despite advancements in surgical resection methods, radio-chemotherapy and newer options as tumour-treating fields and targeted therapies, the median overall survival (OS) of glioblastoma patients remains limited around 20.9 months [2].

Various factors have been identified as prognostic indicators for overall survival, including age, gender, the extent of tumour resection, performance status, MGMT promoter methylation, and temporal muscle thickness [3, 4]. Additionally, treatment intensity [3] and the occurrence of side effects [5] could also have an impact on prognosis. During the course of the standard-of-care Stupp regimen, which consists of combined radio-chemotherapy and adjuvant alkylating chemotherapy with temozolomide, multiple side effects have been documented [6–8]. These include nausea, vomiting, bone marrow suppression, opportunistic infections, fatigue, and liver dysfunction [6–8]. Furthermore, alkylating agents such as temozolomide commonly cause hematological problems such as anaemia, leukopenia, and thrombocytopenia [6–8]. The severity and kind of therapy-related side effects can vary based on factors such as dosage and individual drug metabolism [9]. In GB, only a few studies have been published that focus on the influence of chemotherapy adverse events on prognosis. However, the available data remains inconclusive. For example, one study suggests that prolonged bone marrow suppression is associated with reduced survival rates [10] while another study, that compared combination of CCNU and temozolomide to temozolomide alone, showed no association between occurrence of hematotoxicity and survival [11].

In recent years, nutritional aspects have been increasingly recognized as influencing factors of chemotherapy related toxicity [12]. For example, low albumin levels have been associated with increased toxicity risk, whereas higher body mass index (BMI) appears to be protective in patients with solid tumours [13]. Dietary interventions, such as protein supplementation and short-term fasting, have been shown to possibly play a role to mitigate chemotherapy-induced toxicities in other cancer types, including head and neck, breast, colorectal and esophageal cancers [14–17]. However, data on dietary influence on chemotherapy-related toxicities in glioma patients are limited. Recently, growing evidence also suggests that the gastrointestinal microbiome plays a crucial role in the effectiveness of cytostatic drugs [18, 19]. One way to influence the intestinal microbiota is through dietary interventions [20].

Apart from chemotherapy related side effects, a possible influence of diet on prognosis has been described in patients with cancer [12, 16, 21]. With regard to glioma, several nutritional and inflammatory biomarker scores, such as the *Controlling Nutritional Status* (CONUT) score, the *Prognostic Nutritional Index* (PNI), and hematologic biomarkers such as albumin or fibrinogen have been established to predict OS in glioma patients [22]. Here, higher CONUT or PNI scores indicate poor nutritional status and systemic inflammation and correlate with worse outcomes [22]. However, these scores are based on measurements from the blood and are not able to directly monitor dietary habits, that may themselves influence glioma prognosis.

In summary, data on the influence of dietary habits on the outcome of patients with GB and other gliomas is scarce. To address these gaps, we conducted a retrospective study in a homogeneous cohort of patients with GB. We assessed dietary intake by use of a food frequency questionnaire (FFQ) and correlated nutritional patterns with chemotherapy-associated toxicities and overall survival.

## Methods

### Study cohort

This study was designed as a retrospective longitudinal analysis with prospective data collection. It included all patients with histological diagnosis of GB who received treatment at the certified Neuro-oncology Center Regensburg between January 2010 and December 2019. Other inclusion criteria were age of at least 18 years at time of diagnosis and consent to complete the questionnaires. We retrieved patient and general practitioner (GP) contact information from the cancer registry of the University Hospital Regensburg. Following data collection, the data underwent a two-step pseudonymization process and was stored locally in a secure manner in accordance with European Union and local data protection regulations.

Exclusion criteria were missing information in the FFQ, insufficient clinical information during the course of first line therapy to assess side effects and other treatment than standard therapy with combined radiotherapy and adjuvant chemotherapy with temozolomide.

We selected the initial cohort of patients in 09/2020. Informed consent was obtained from 09/2020 to 04/2021 and questionnaires were distributed immediately after consent. Between 05/2021 and 06/2023 we extracted clinical information from medical records.

### Data acquisition

The collection of baseline data is described in detail in a previous publication which focused on antibiotic use and prognosis in patients with GB [23]. The collected data included tumour histology and genetics, as well as patient demographics and prognostic factors. Additionally, clinical information was collected, including therapy regimens and their duration, treatment-related side effects during the course of radio-chemotherapy which were named in medical reports, BMI and comorbidities.

We used a newly developed questionnaire to assess information on dietary habits in general and a 35-item FFQ to query data on intake of specific foods. The questionnaires were completed by GB patients or by their close family members if the patients were already deceased or not capable of answering the questions by themselves.

We collected information on number and timepoints of meals per day, fluctuations of weight within 12 months before the time of GB diagnosis, inflammatory gastrointestinal diseases, food intolerances or avoided food, use of dietary supplements or vitamins and a self-evaluation of dietary habits. In our FFQ, we assessed the following single items: meat, sausages, poultry, fish, seafood, potatoes, pasta, rice, soy products or tofu, raw vegetables, cooked vegetables, fresh fruits, fast food, ready meals, white or mixed bread, whole wheat bread, oatmeal or muesli, yoghurt, milk, cheese, eggs, chips, chocolate, other sweets, cake or pastries, juice, soft drinks, only water, beer, wine, distilled alcoholic beverages, coffee, black tea, fruit tea, sugar for coffee and tea. Categories of food frequencies of the FFQ were “never”, “<1x/month”, “1-3x/month”, “1-2x/week”, “3-6x/week”, “daily” and “several times a day”.

### Calculation of the dietary score

Each food item was recoded into a score of 0 (poor adherence to Mediterranean style diet), 1 (moderate adherence to Mediterranean style diet), or 2 (good adherence to Mediterranean style diet) based on recommendations of the German Nutrition Society (Deutsche Gesellschaft für Ernährung, DGE, https://www.dge.de), which align with a Mediterranean-style diet [24]. This kind of diet is characterized by high intake of fruits, vegetables and other plant-based foods and moderate intake of fish, white meat and dairy products [24]. The implemented scoring matrix for every single item is made available in the supplement. Missing values and non-responses were excluded from calculations. The individual food scores were then summed up to generate a raw total dietary score. To account for variations in the number of valid responses per participant, the raw total score was adjusted for the number of valid responses.

For further analyses, patients were divided into two groups based on the median of the adjusted dietary score. All further comparisons were conducted between these two groups to assess potential differences in dietary patterns and other study variables. Because some categories were chosen only rarely, we simplified the FFQ categories into 3 groups, namely “<1x/week”, “1-6x/week” and “>=1x/day” for presentation of differences of single FFQ items between the dietary score groups and calculation of cox regression analysis.

### Clinical endpoints

Clinical data, blood counts and side effects were documented. However, due to insufficient data on the severity of clinical side effects, it was not feasible to categorize them according to Common Terminology Criteria for Adverse Events v5.0 (CTCAE) grades.

Clinical outcome endpoints were occurrence of any side effect, myelodepression, infection (unspecified), cutaneous side effects, nausea, vomiting, diarrhoea, obstipation, loss of appetite, vertigo, headache, fatigue and alopecia.

Survival-related endpoints were overall survival (OS), calculated as time from diagnosis to death and progression-free survival (PFS), calculated as time from diagnosis to disease progression or death.

### Statistical analyses

All analyses were conducted using IBM SPSS Statistics, version 29. The Pearson chi-square test was employed to assess the independence between categorical variables and dietary score groups. Mean comparisons were performed using the Student’s t-test and the Mann-Whitney U-test.

Associations between nutrition score, side effects, and lab value changes were examined using univariable and multivariable binary logistic regression. A p-value of less than 0.05 was considered statistically significant.

Kaplan-Meier analyses and multivariable Cox regression analyses were performed to estimate overall survival. Survival curves were compared via log-rank tests.

We adjusted multivariable binary logistic regression analyses and cox regression analyses for demographic and prognostic factors that may affect clinical outcomes. These variables were selected a priori and included age, sex, BMI, prior independent tumour disease, MGMT promoter methylation status, IDH mutational status, Karnofsky performance score, and extent of resection. Adjustment for BMI was included to account for potential confounding related to nutritional status.

## Results

### Study cohort

**Fig. 1:**
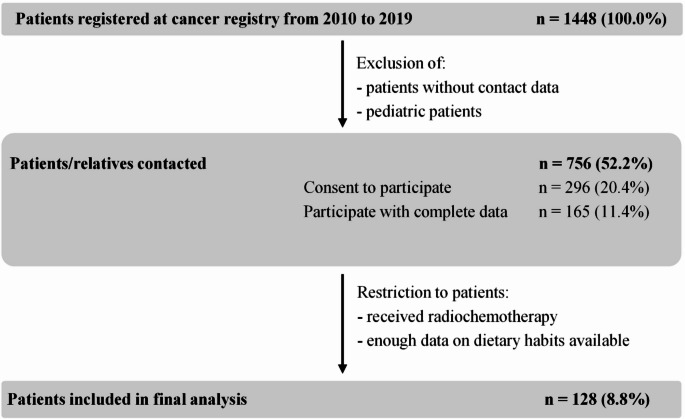
Consort flow chart of patients with prospective data collection

We identified 1,448 glioma patients at the local cancer registry database, who were treated at the certified Neuro-oncology Center in Regensburg from 2010 to 2019. After exclusion of pediatric patients, patients that were treated primarily at another hospital and patients with missing contact information, 756 patients (52.2%) were suitable for inclusion in this study. We received informed consent from 296 patients or their close family members (20.4%). However, food frequency questionnaires with enough information for further analyses and sufficient clinical information was available only from 165 patients (11.4%). We further restricted our main analysis to patients who received the standard therapy according to the Stupp regimen [6]. Consequently, we included 128 patients (8.8%) in our final analysis (Fig. 1).

The median age of patients in our cohort was 59.1 years (25.8–83.4) and there were fewer females (39.8%) than males (60.2%) (see Table 1 for details on overall baseline characteristics).

We calculated dietary scores for every patient as described in the methods section and then divided the study cohort into two groups according to the median dietary score. 65 patients (50.8%) had a dietary score of 35 or more, while 63 (49.2%) had a score of less than 35. There was a higher proportion of females in the higher score groups (49.2% vs. 30.2%, *p* = 0.028). However, there were no significant differences regarding age, BMI, Karnofsky performance status, IDH1 mutational status, MGMT promotor methylation and antibiotic use before the time of diagnosis between the two groups (Table 1).

**Table 1:** Patient characteristics dependent on dietary score group

		Dietary score									
		Higher Score (35+)			Lower Score (< 35)			total			
		*n*	%		*n*	%		*n*		%	*p* value
total		65	100%		63	100%		128		100%	
**Sex**	female	32	49,2%		19	30,2%		51		39.8%	**0.028**
	male	33	50,8%		44	69,8%		77		60.2%	
**Age at time of diagnosis**	< 50,0	9	13,8%		11	17,5%		20		15.6%	0.466
	50,0–59,9	23	35,4%		28	44,4%		61		39,8%	
	60,0–69,9	19	29,2%		16	25,4%		35		27,3%	
	>=70,0	14	21,5%		8	12,7%		22		17,2%	
**BMI**	< 25,0	23	35,4%		14	22,2%		37		28,9%	0.386
	25,0–29,9	21	32,3%		25	39,7%		46		35,9%	
	>=30,0	14	21,5%		14	22,2%		28		21,9%	
	unknown	7	10,8%		10	15,9%		17		13,3%	
**Karnofsky**	< 70	5	7,7%		0	0,0%		5		3,9%	0.106
	70	6	9,2%		7	11,1%		13		10,2%	
	80	9	13,8%		8	12,7%		17		13,3%	
	90	25	38,5%		23	36,5%		48		37,5%	
	100	17	26,2%		15	23,8%		32		25,0%	
	unknown	3	4,6%		10	15,9%		13		10,2%	
**IDH1 mutational status**	wildtype	54	83,1%		50	79,4%		104		81,3%	0.643
	mutation	2	3,1%		1	1,6%		3		2,3%	
	unknown	9	13,8%		12	19,0%		21		16,4%	
**MGMT promoter methylation status**	yes	29	44,6%		27	42,9%		56		43,8%	0.940
	no	29	44,6%		28	44,4%		57		44,5%	
	unknown	7	10,8%		8	12,7%		15		11,7%	
**Previous other tumour**	yes	6	9,2%		5	7,9%		11		8,6%	0.794
	no	59	90,8%		58	92,1%		117		91,4%	
**Extent of resection**	complete	25	38,5%		28	44,4%		53		41,4%	0.387
	partial	31	47,7%		24	38,1%		55		43,0%	
	biopsy	9	13,8%		9	14,3%		18		14,1%	
	unknown	0	0,0%		2	3,2%		2		1,6%	
**Antibiotic use before diagnosis**	yes	7	10,8%		10	15,9%		17		13,3%	0.308
	no	28	43,1%		32	50,8%		60		46,9%	
	unknown	30	46,2%		21	33,3%		51		39,8%	

### Evaluation of dietary habits between the dietary score groups

Next, we examined how dietary habits, assessed through self-evaluation and food items from the food frequency questionnaire, varied between the two dietary score groups.

Together, 58 patients (89.2%) in the higher score group had 3–4 meals per day compared to 48 patients (76.2%) in the lower score group. Of note, weight fluctuations occurred less often in patients with a score of 35 or more (12.3% vs. 27.0%). However, both observations were not statistically significant (Table 2). Sixty-two patients (95.4%) in the higher score group classified their dietary habits as balanced, while this was only the case in 49 patients (77.8%) in the lower score group (*p* = 0.003).

From the 35 food items that were used to calculate the score, only the frequency of intake of sausages (*p* = 0.039), fish (*p* = 0.037), potatoes (*p* = 0.022), fruits (p = < 0.001), ready meals (*p* = 0.003), whole wheat bread (p = < 0.001), oatmeal/muesli (p = < 0.001), milk (*p* = 0.006), cheese (*p* = 0.030), softdrinks (p = < 0.001), water (p = < 0.001), beer (*p* = 0.009) and sugar for tea/coffee (p = < 0.001) were significantly different between the two groups (see Supplement for details).

**Table 2:** Different dietary habits between the dietary score groups

				Dietary score				
		Higher Score (35+)				Lower Score (< 35)		
		*n* = 65	%			*n* = 63	%	p value
**Meals per day**	1–2	3	4,6%			9	14,3%	0.170
	3–4	58	89,2%			48	76,2%	
	5–6	4	6,2%			5	7,9%	
	> 6	0	0,0%			1	1,6%	
**Balanced diet**	yes	62	95,4%			49	77,8%	0.003
	no	3	4,6%			14	22,2%	
**Low carb diet**	yes	3	4,6%			1	1,6%	0.325
	no	62	95,4%			62	98,4%	
**Low fat diet**	yes	10	15,4%			8	12,7%	0.662
	no	55	84,6%			55	87,3%	
**High-protein diet**	yes	9	13,8%			9	14,3%	0.943
	no	56	86,2%			54	85,7%	
**Weight fluctuations**	yes	8	12,3%			17	27,0%	0.110
	no	56	86,2%			45	71,4%	
	no information	1	1,5%			1	1,6%	
**Food intolerances**	yes	4	6,2%			4	6,3%	0.613
	no	60	92,3%			59	93,7%	
	no information	1	1,5%			0	0,0%	
**Use of nutritional supplements**	yes	11	16,9%			12	19,0%	0.754
	no	54	83,1%			51	81,0%	

### Chemotherapy-related toxicity

Then, we investigated if the dietary score was associated with the risk of occurrence of chemotherapy-related side effects (Table 3). We found a higher number of patients with infection (13.8% vs. 3.2%, *p* = 0.031) and obstipation (7.7% vs. 0%, *p* = 0.025) in the higher score group. We also observed a trend for higher occurrence of myelodepression (32.3% vs. 17.5%, *p* = 0.052) and headache (26.2% vs. 14.3%, *p* = 0.095) in this group.

In our multivariable binary logistic regression analysis model, a dietary score of 35 or more was a significant predictor for occurrence of infection (OR = 12.33, 95% CI: 1.36-111.98; *p* = 0.026) and vertigo (OR = 0.21, 95% CI: 0.05–0.85; *p* = 0.029). All other side effects were not significantly associated with the dietary score (Table 3).

**Table 3:** Toxicity during adjuvant radiochemotherapy

	Dietary score					Multivariable binary logistic regression analysis*		
	Score 35+ (*n* = 65)		Score < 35 (*n* = 63)					
	*n*	%	*n*	%	*p* value (Chi²)		OR (95% CI)	*p* value
Any toxicity/side effect	50	76,9%	42	66,7%	0.197		1.30 (0.48–3.50)	0.610
Myelodepression	21	32,3%	11	17,5%	0.052		1.58 (0.54–4.61)	0.401
Infection	9	13,8%	2	3,2%	**0.031**		**12.33 (1.36-111.98)**	**0.026**
Cutaneous side effects	5	7,7%	4	6,3%	0.766		0.55 (0.06–4.74)	0.584
Nausea	21	32,3%	22	34,9%	0.754		0.73 (0.28–1.87)	0.505
Vomitting	10	15,4%	7	11,1%	0.476		1.59 (0.45–5.65)	0.470
Diarrhea	3	4,6%	3	4,8%	0.969		1.81 (0.08–41.49)	0.710
Obstipation	5	7,7%	0	0,0%	**0.025**		**	**
Loss of appetite	5	7,7%	6	9,5%	0.712		0.56 (0.04–7.61)	0.659
Vertigo	7	10,8%	12	19,0%	0.188		**0.21 (0.05–0.85)**	**0.029**
Headache	17	26,2%	9	14,3%	0.095		2.19 (0.70–6.88)	0.180
Fatigue	39	60,0%	31	49,2%	0.220		1.30 (0.56–3.02)	0.543
Alopecia	14	21,5%	8	12,7%	0.185		1.71 (0.49–5.91)	0.399

*adjustment for age, sex, BMI, prior independent tumour disease, MGMT promoter methylation status, IDH mutational status, Karnofsky performance score, and extent of resection

** not available due to low case number

### Survival analysis

**Fig. 2:**
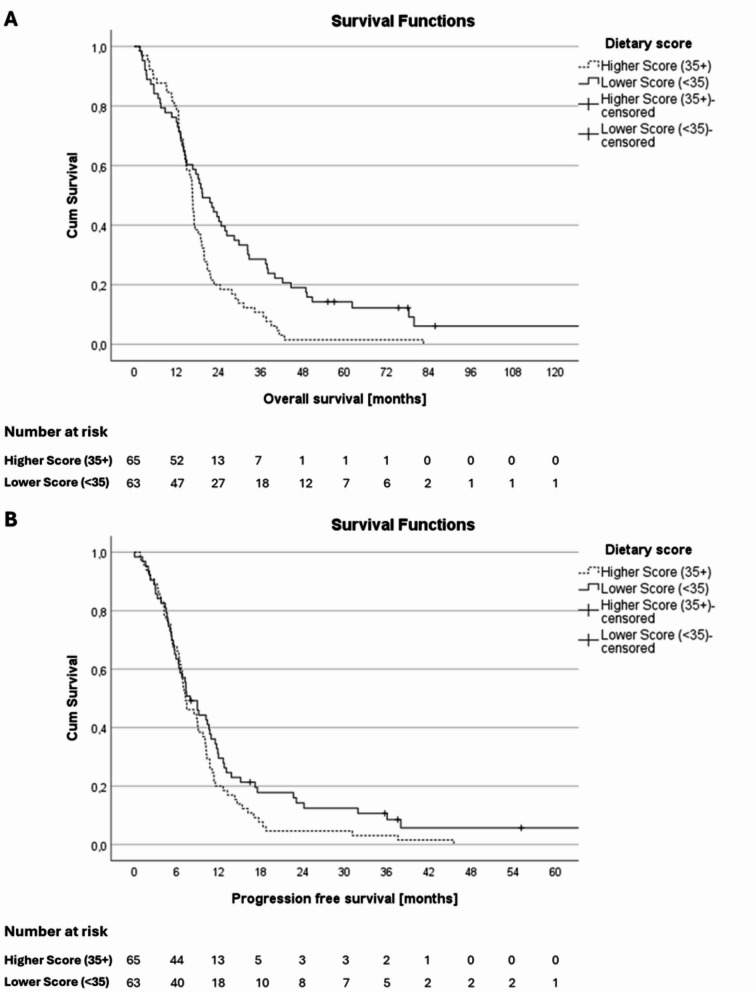
Overall survival (OS) and Progression free survival (PFS) of patients with higher dietary scores compared to lower dietary scores. **A** Median OS of the higher score group was worse (16.6 months vs. 19.4 months, *p* = 0.004). This finding was confirmed by multivariable cox regression analysis (HR 1.60, 95% CI 1.04–2.46; *p* = 0.034). **B** PFS was not significantly different between both groups with 7.3 months vs. 8.0 months (HR 1.31, 95% CI 0.86–1.98; *p* = 0.205)

We then investigated, if a higher dietary score had an influence on median overall survival. Within our cohort of 128 patients, 122 patients (95.3%) died during the observation period. All patients that did not die within the study period were in the lower score group. Median survival in the higher score group was 16.6 months, compared to 19.4 months in the lower score group (*p* = 0.004) with a clear and sustained separation of Kaplan Meier curves (Fig. 2). Furthermore, two year- and five year-survival rates were worse in patients with higher dietary scores with 20.0% vs. 42.9% (*p* = 0.005) and 1.5% vs. 11.1% (*p* = 0.025) respectively. Multivariable cox regression analysis also indicated worse survival in patients with dietary scores of 35 or more (HR 1.70, 95% CI 1.10–2.62; *p* = 0.017; please refer to the suppl. Table 3 for HR and 95% CIs of all variables in the full model). Median PFS was not significantly different between both groups with 7.3 months vs. 8.0 months (HR 1.31, 95% CI 0.86–1.98; *p* = 0.205).

### Sensitivity analyses

We conducted a sensitivity analysis excluding all patients that were already deceased at the time of data collection in order to exclude proxy responses. In this subset, 7 patients belonged to the higher dietary score group and 13 to the lower score group. These patients were different from the cohort of the main analysis as 17 (85%) had MGMT promotor methylation and 16 (80%) had undergone complete resection during first line treatment. Despite the low case numbers, the higher score group continued to show significantly worse survival, with a median overall survival of 28.7 months compared to 62.1 months in the lower score group (*p* = 0.012). This was confirmed by multivariable cox regression analysis, with patients with higher dietary scores showing worse survival (HR 3.62, 95% CI 1.24–10.6; *p* = 0.019).

Next, we excluded all patients with IDH mutation from the analysis. With regard to the baseline patient characteristics, there were still less females (29.0%) in the lower dietary score group. When we compared occurrence of adverse effects during the course of radio-chemotherapy between higher vs. lower score groups, infection occurred in 9 (14.3%) vs. 2 (3.2%) patients (*p* = 0.029), obstipation in 5 (7.9%) vs. 0 (0%) patients (*p* = 0.0024) and myelodepression in 21 (33.3%) vs. 11 (17.7%) patients (*p* = 0.046). Median overall survival was 16.7 vs. 19.3 months (*p* = 0.007). Patients with higher dietary scores still had worse survival in multivariable cox regression (HR 1.59, 95% CI 1.01–2.49; *p* = 0.045).

## Discussion

In this retrospective cohort study, we present data on dietary habits in relation to toxicity of chemotherapy and overall survival of patients with glioblastoma. We detected significant differences in food item intake that well discriminated the two groups with a high and low dietary score. Surprisingly, a higher Mediterranean-like dietary score was linked to a higher rate of certain adverse events during chemotherapy and shorter median survival (16.6 vs. 19.4 months), confirmed by multivariable Cox regression.

In view of demographic factors, our study shows a higher rate of females in the higher dietary score group, which represents a healthier diet according to the German Nutrition Society recommendations. This fits well with published literature, as females are known to keep a presumably healthier diet than males [25], which underscores the validity of our data. All other demographic, prognostic and outcome factors in our study mirror a typical GB population.

Some other published studies aim at establishing a link between nutrition and glioma. However, most of these studies focused on associations with the risk of glioma [26–36], which may modify the incidence, but not treatment effects in GB. In a series of studies, the authors found an inverse association with risk of glioma after consumption of legume and nuts [29], plant-based diet [30], Mediterranean diet [31] fiber and vitamins [34]. Contrary, an increased risk was observed in patients with Western diet [32], and high rice consumption [33]. The role of meat consumption thereby remains unclear with inconsistent results [34–36]. Data on possible associations between dietary habits and chemotherapy related toxicity and prognosis in patients with glioma remains scarce.

When evaluating side effects of chemotherapy in relation to our dietary score, we observed a significantly higher occurrence of infection and obstipation. Though infection encompasses all types, the majority likely are respiratory or urinary tract infections. Patients with a low-score diet tend to have a higher caloric intake which could lead to lower risk of sarcopenia. We speculate that this could be part of an explanation as temporal muscle thickness as a surrogate marker of sarcopenia has prognostic relevance in patients with glioblastoma [4]. The observed trend towards higher occurrence of myelodepression and headaches should not be overinterpreted, as patient numbers were small. To our knowledge, there are no other studies in patients with glioma that addressed this question. Nonetheless, there is some data on other entities. In general, low albumin levels are associated with a higher risk of toxicity, while higher BMI of more than 30 seems to be protective [13]. Although adherence to a Mediterranean diet is inversely associated with BMI [37], BMI was not significantly different between the dietary score groups in our study. Low protein intake was shown to lead to more toxicity in patients with neck, colorectal, and oesophageal cancer [14, 16, 17]. In patients with cervical cancer, high fiber diets lead to reduced gastrointestinal toxicity during radiotherapy [38]. In our study, the higher score group especially showed higher consumption of fish, potatoes, vegetables, fruits, whole wheat bread, oatmeal and dairy products (curd, yoghurt, milk, cheese).

In our cohort, patients with a lower dietary score showed an increased overall survival that was consistently seen in Kaplan Meier- and multivariable Cox regression analysis. We found no other studies, that investigated food frequency data with regard to survival in patients with glioblastoma. However, there are small studies which showed a potentially beneficial role of ketogenic diets in patients with glioma [39]. This dietary approach, which is characterized by very low carbohydrate intake, aims to induce ketone body production as an alternative energy source. This is based on the assumption that glioma cells rely primarily on glucose for their metabolism and are not capable of metabolizing ketone bodies [39]. However, patients with a low score tended to consume more foods that are considered unhealthy, such as chips, sweets, cake, soft drinks, and alcoholic beverages. These items are not permitted on a ketogenic diet and are typically high in calories. In contrast, foods associated with higher scores included vegetables, fruits, dairy products, and water.

In other cancers, adherence to a Mediterranean diet has been associated with improved survival, as summarized in a meta-analysis, however with low evidence overall [40]. These benefits have been attributed to high fibre intake, consumption of phytochemicals with anticancer properties, reduced exposure to harmful compounds such as N-nitroso compounds found in processed meats and positive modulation of the immune system [40]. Positive effects were observed primarily in patients with colorectal cancer, gastric, bladder or lung cancer [40], that either directly involve the gastrointestinal tract or are highly vascularized. GB, on the other hand, is considered a immunologically “cold” tumour with a highly immunosuppressive microenvironment [41]. Nonetheless, there are also conflicting results. Contrary to our results, in other cancer entities such as breast cancer, mediterranean diet has been associated with better prognosis [42]. In contrast, dietary scores based on the American Cancer Society recommendations did not show a significant association with improved breast cancer survival [43].

While to our knowledge there is no data on direct associations between food items and mortality in patients with glioma, various nutritional and inflammatory biomarker scores, including the CONUT score, the PNI, as well as hematological markers like albumin and fibrinogen, have been linked to worse outcomes in glioma patients (Morelli et al., 2025). Elevated CONUT and PNI scores reflect poor nutritional status and heightened systemic inflammation. However, these scores rely on blood-based biomarkers and not on dietary habits. We were not able to adjust for such biomarker scores because we had no records of albumin measurements. We therefore used BMI as a surrogate for nutritional status in our multivariable models.

One possible explanation, how diet could affect toxicity and prognosis of cancer is through modulation of the intestinal microbiota. A higher microbial diversity has been associated with less toxicity of radio-chemotherapy in patients with cervical cancer [44]. Response and toxicity of chemotherapy, especially immune checkpoint inhibition was also shown to be dependent of microbiota [45]. Interestingly, the interactions are not always as expected. For example, use of probiotics lead to even less response to immune therapy in patients with melanoma [46]. It remains open if differences in microbiota composition which could be shaped by food intake may contribute to the prognostic differences in our study, as we did not control for microbiota composition.

Our study has several limitations. A major limitation is, that some of the questionnaires were collected from close relatives, as most patients were already deceased. Therefore, we could not use a validated food frequency questionnaire. In some cases, the questionnaires were completed several months or even years after diagnosis, which may have introduced recall bias. Another potential limitation is the retrospective design, which may introduce selection bias, especially since there is a high number of proxy respondents for deceased patients. However, patients were registered sequentially, and predefined selection criteria were consistently applied to the initial cohort. Furthermore, most patients were already deceased when FFQ when information was collected with FFQs. Because we lacked information on who answered the questionnaires, we were unable to perform a sensitivity analysis excluding potential proxy responses. The retrospective design may have introduced bias in survival analyses due to potential group imbalances. However, multivariable models adjusted for possible confounders and still revealed significant differences. Finally, diagnosis of GB included some patients with IDH mutation, in accordance with the former WHO classification but we may have missed molecular glioblastomas. Both could affect overall survival. We therefore included IDH status in the multivariable model. Furthermore, we performed a sensitivity analysis with exclusion of glioma patients with IDH mutation which showed comparable results. Finally, due to the retrospective design of our study, we were not able to infer causality. The results of our study therefore need to be prospectively validated.

Our study also has important strengths. This is the first study in patients with histologically diagnosed glioblastoma that focused on the importance of dietary habits with regard to chemotherapy-related toxicity and overall survival in glioma patients. Our initial cohort included the high number of 1,448 patients, which was strictly selected using predefined inclusion criteria to obtain a highly homogeneous final cohort with high-quality data that were obtained from certified or accredited databases within the cancer registry and institutional records. We therefore believe that our data are valid and suited to generate strong hypotheses for future research.

In summary, using a dietary score based on food frequency questionnaire responses and implementing the recommendations of the German Nutrition Society, we found that dietary adherence to the recommendations, comparable to a Mediterranean diet, correlated with worse survival. These findings underscore the potential impact of nutrition on cancer treatment outcomes. It is not possible to infer causality due to the retrospective design of our study. Prospective cohort studies or interventional trials are necessary to validate the findings.

## Electronic supplementary material

Below is the link to the electronic supplementary material.

Supplementary Material 1: **S1** Questionnaire of dietary habits and FFQ

Supplementary Material 2: **S2** Scoring matrix

Supplementary Material 3: **S3** Multivariable Cox Regression– full model

Supplementary Material 4: **S4** Frequency of single food items between the dietary score groups

## Data Availability

Anonymized data supporting the findings of this study, along with relevant SPSS or R code used in the analysis, are available from the corresponding author upon reasonable request, subject to institutional review and applicable data use agreements.
