# Supplementary material for: Dietary habits in relation to outcome and therapy-related toxicity in patients with glioblastoma – a retrospective cohort study
Source: J Neurooncol. 2025 Jul 21;175(1):345–55. doi: 10.1007/s11060-025-05137-3 (PMC12367922; doi:10.1007/s11060-025-05137-3)
Supplement: Supplementary file 1 — Supplementary Material 1: S1 Questionnaire of dietary habits and FFQ [file 11060_2025_5137_MOESM1_ESM.docx]

Questionnaire of dietary habits

The following questions apply to the last 12 months before time of diagnosis of the brain tumor.

How many meals did you have per day?

**Please select just one!**

□ 1-2 □ 3-4 □ 5-6 □ >6

Which of the following meals did you usually take?

**Please select all applicable answers!**

□ breakfast □ lunch □ dinner □ snacks

Which of the following terms describes your dietary style best?

**Please select all applicable answers!**

□ balanced □ vegetarian □ vegan □ low-carb

□ low-fat □ high-protein

□ other: ___________________________________________________________________________

Please indicate your weight approximately 12 months before diagnosis: ________kg

Did you experience weight fluctuations in the 12 months before diagnosis?

□ no

□ yes, : □ gain of weight _______ kg □ loss of weight _______ kg

Were there any inflammatory diseases or infections of the gastrointestinal tract??

□ no

□ yes: □ Crohn’s disease □ ulcerative colitis □ Irritable bowel syndrome

□ Gastritis □ gastric ulcer

treatment: _______________________________________________________________________

Food intolerances/allergies:

□ no

□ yes: □ Celiac disease □ Gluten intolerance □ Lactose intolerance

□ Fructose intolerance □ Histamine intolerance

□ other: _____________________________________________________________________________

Were any specific foods avoided?

□ no

□ yes: ___________________________________________________________________________

Intake of dietary supplements/probiotics/vitamin supplements/protein supplements?

□ no

□ yes: ___________________________________________________________________________

If yes, how often? □ >=1x/d □ 1x/d □ 3-6x/week □1-2x/week □1-3x/month □ <1x/month

How often were the following foods usually consumed?

|  | never | Less than 1x/ month | 1-3x/ month | 1-2x/ week | 3-6x/ week | daily | >=1x/d |
| --- | --- | --- | --- | --- | --- | --- | --- |
| Meat |  |  |  |  |  |  |  |
| Sausages |  |  |  |  |  |  |  |
| Poultry |  |  |  |  |  |  |  |
| Fish |  |  |  |  |  |  |  |
| Seafood |  |  |  |  |  |  |  |
| Potatoes |  |  |  |  |  |  |  |
| Pasta |  |  |  |  |  |  |  |
| Rice |  |  |  |  |  |  |  |
| Soy products or tofu |  |  |  |  |  |  |  |
| Raw vegetables |  |  |  |  |  |  |  |
| Cooked cegetables |  |  |  |  |  |  |  |
| Fresh frutis |  |  |  |  |  |  |  |
| Fast food |  |  |  |  |  |  |  |
| Ready meals |  |  |  |  |  |  |  |
| White or mixed bread |  |  |  |  |  |  |  |
| Whole wheat bread |  |  |  |  |  |  |  |
| Oatmeal or muesli |  |  |  |  |  |  |  |
| Yoghurt |  |  |  |  |  |  |  |
| Milk |  |  |  |  |  |  |  |
| Cheese |  |  |  |  |  |  |  |
| Eggs |  |  |  |  |  |  |  |
| Chips |  |  |  |  |  |  |  |
| Chocolate |  |  |  |  |  |  |  |
| Other sweets |  |  |  |  |  |  |  |
| Cake or pastries |  |  |  |  |  |  |  |
| Juice |  |  |  |  |  |  |  |
| Soft drinks |  |  |  |  |  |  |  |
| Only water |  |  |  |  |  |  |  |
| Beer |  |  |  |  |  |  |  |
| Wine |  |  |  |  |  |  |  |
| Distilled alcoholic beverages |  |  |  |  |  |  |  |
| Coffee |  |  |  |  |  |  |  |
| Black tea |  |  |  |  |  |  |  |
| Fruit tea |  |  |  |  |  |  |  |
| Sugar for coffee and tea |  |  |  |  |  |  |  |

Were any specific foods avoided after diagnosis?

□ no

□ yes: _______________________________________________________________________

Were any specific foods consumed more frequently after diagnosis?

□ no

□ yes: _______________________________________________________________________

Was the diet changed in any other way after diagnosis?

□ no

□ yes: _______________________________________________________________________
