# Supplementary material for: Dietary habits in relation to outcome and therapy-related toxicity in patients with glioblastoma – a retrospective cohort study
Source: J Neurooncol. 2025 Jul 21;175(1):345–55. doi: 10.1007/s11060-025-05137-3 (PMC12367922; doi:10.1007/s11060-025-05137-3)
Supplement: Supplementary file 2 — Supplementary Material 2: S2 Scoring matrix [file 11060_2025_5137_MOESM2_ESM.docx]

Scoring matrix for food frequency questionnaire items

|  | never | <1x/ month | 1-3x/ month | 1-2x/ week | 3-6x/ week | daily | several times a day |
| --- | --- | --- | --- | --- | --- | --- | --- |
| Meat | 0 | 1 | 2 | 2 | 1 | 0 | 0 |
| Sausages | 0 | 1 | 2 | 2 | 1 | 0 | 0 |
| Poultry | 0 | 1 | 2 | 2 | 1 | 0 | 0 |
| Fish | 0 | 1 | 2 | 2 | 1 | 0 | 0 |
| Seafood | 0 | 1 | 2 | 2 | 1 | 0 | 0 |
| Potatoes | 0 | 0 | 0 | 0 | 1 | 2 | 1 |
| Pasta | 0 | 0 | 0 | 0 | 1 | 2 | 1 |
| Rice | 0 | 0 | 0 | 0 | 1 | 2 | 1 |
| Soya products/Tofu | 0 | 0 | 0 | 1 | 2 | 2 | 1 |
| Raw vegetables | 0 | 0 | 0 | 0 | 1 | 2 | 2 |
| Cooked vegetables | 0 | 0 | 0 | 0 | 1 | 2 | 2 |
| Fresh fruits | 0 | 0 | 0 | 0 | 1 | 2 | 2 |
| Fast food | 2 | 2 | 1 | 1 | 0 | 0 | 0 |
| Ready meals | 2 | 2 | 1 | 1 | 0 | 0 | 0 |
| White or mixed bread | 0 | 0 | 0 | 0 | 1 | 2 | 2 |
| Whole wheat bread | 0 | 0 | 0 | 0 | 1 | 2 | 2 |
| Oatmeal/ muesli | 0 | 0 | 0 | 1 | 1 | 2 | 1 |
| Yogurt | 0 | 0 | 0 | 1 | 1 | 2 | 2 |
| Milk | 0 | 0 | 0 | 1 | 1 | 2 | 2 |
| Cheese | 0 | 0 | 0 | 1 | 1 | 2 | 1 |
| Eggs | 2 | 2 | 2 | 2 | 1 | 0 | 0 |
| Chips, pretzel sticks | 2 | 2 | 1 | 1 | 0 | 0 | 0 |
| Chocolate | 2 | 2 | 1 | 1 | 0 | 0 | 0 |
| Other sweets | 2 | 2 | 1 | 1 | 0 | 0 | 0 |
| Cake, pastries | 2 | 2 | 1 | 1 | 0 | 0 | 0 |
| Juice | 2 | 2 | 1 | 1 | 0 | 0 | 0 |
| Soft drinks | 2 | 2 | 1 | 1 | 0 | 0 | 0 |
| Only water | 0 | 0 | 0 | 0 | 0 | 1 | 2 |
| Beer | 2 | 2 | 1 | 1 | 0 | 0 | 0 |
| Wine | 2 | 2 | 1 | 1 | 0 | 0 | 0 |
| High-proof alcoholic drinks | 2 | 2 | 1 | 1 | 0 | 0 | 0 |
| Coffee | 0 | 0 | 0 | 0 | 0 | 1 | 2 |
| Black tea | 0 | 0 | 0 | 0 | 0 | 1 | 2 |
| Fruit tea | 0 | 0 | 0 | 0 | 0 | 1 | 2 |
| Sugar for Coffee/Tea | 2 | 2 | 1 | 1 | 0 | 0 | 0 |
