# Supplementary material for: Dietary habits in relation to outcome and therapy-related toxicity in patients with glioblastoma – a retrospective cohort study
Source: J Neurooncol. 2025 Jul 21;175(1):345–55. doi: 10.1007/s11060-025-05137-3 (PMC12367922; doi:10.1007/s11060-025-05137-3)
Supplement: Supplementary file 3 — Supplementary Material 3: S3 Multivariable Cox Regression– full model [file 11060_2025_5137_MOESM3_ESM.docx]

|  | | | | |
| --- | --- | --- | --- | --- |
| **Multivariable cox regression** | | | | |
|  | **P value** | **HR** | **95,0% CI** | |
|  |  |  | **Lower** | **Upper** |
| Dietary score (high vs. low) | ,017 | 1,697 | 1,098 | 2,623 |
| sex | ,135 | 1,397 | ,901 | 2,166 |
| Age at time of diagnosis | ,212 |  |  |  |
| Age at time of diagnosis (1) | ,290 | 1,374 | ,763 | 2,474 |
| Age at time of diagnosis (2) | ,051 | 1,928 | ,996 | 3,732 |
| Age at time of diagnosis (3) | ,125 | 1,767 | ,853 | 3,658 |
| BMI at time of diagnosis | ,472 |  |  |  |
| BMI at time of diagnosis (1) | ,374 | ,790 | ,471 | 1,327 |
| BMI at time of diagnosis (2) | ,354 | ,753 | ,413 | 1,372 |
| BMI at time of diagnosis (3) | ,137 | ,487 | ,189 | 1,257 |
| Karnofsky at time of diagnosis | <,001 |  |  |  |
| Karnofsky at time of diagnosis (1) | ,098 | ,394 | ,131 | 1,188 |
| Karnofsky at time of diagnosis (2) | ,053 | ,338 | ,112 | 1,015 |
| Karnofsky at time of diagnosis (3) | ,041 | ,350 | ,128 | ,957 |
| Karnofsky at time of diagnosis (4) | <,001 | ,150 | ,051 | ,440 |
| Karnofsky at time of diagnosis (5) | ,766 | 1,242 | ,297 | 5,192 |
| IDH1 mutation | ,247 |  |  |  |
| IDH1 mutation (1) | ,220 | 2,182 | ,627 | 7,600 |
| IDH1 mutation (2) | ,276 | ,713 | ,388 | 1,311 |
| MGMT | <,001 |  |  |  |
| MGMT (1) | <,001 | ,268 | ,164 | ,439 |
| MGMT (2) | ,053 | ,472 | ,221 | 1,010 |
| Previous tumor disease | ,777 | ,899 | ,429 | 1,882 |
| Extent of resection | <,001 |  |  |  |
| Extent of resection (1) | ,010 | 1,875 | 1,159 | 3,034 |
| Extent of resection (2) | <,001 | 3,909 | 2,057 | 7,429 |
| Extent of resection (3) | ,340 | 2,275 | ,421 | 12,308 |
| In first therapy | . |  |  |  |
