# Supplementary material for: Dietary habits in relation to outcome and therapy-related toxicity in patients with glioblastoma – a retrospective cohort study
Source: J Neurooncol. 2025 Jul 21;175(1):345–55. doi: 10.1007/s11060-025-05137-3 (PMC12367922; doi:10.1007/s11060-025-05137-3)
Supplement: Supplementary file 4 — Supplementary Material 4: S4 Frequency of single food items between the dietary score groups [file 11060_2025_5137_MOESM4_ESM.docx]

**Supplemental Table: Frequency of single food items between the dietary score groups (only results with p<0.100 shown)**

|  | |  | | **Dietary score** | | |  |
| --- | --- | --- | --- | --- | --- | --- | --- |
|  |  | Higher Score (35+) | |  | Lower Score (<35) | |  |
|  |  | n=65 | % |  | n=63 | % | p value |
| **Sausages** | <1x/w | 7 | 11,1% |  | 5 | 7,9% | 0.039 |
|  | 1-6x/w | 44 | 69,8% |  | 33 | 52,4% |  |
|  | >=1x/d | 12 | 19,0% |  | 25 | 39,7% |  |
| **Fish** | <1x/w | 32 | 51,6% |  | 44 | 69,8% | 0.037 |
|  | 1-6x/w | 30 | 48,4% |  | 19 | 30,2% |  |
|  | >=1x/d | 0 | 0,0% |  | 0 | 0,0% |  |
| **Potatoes** | <1x/w | 1 | 1,6% |  | 7 | 11,1% | 0.022 |
|  | 1-6x/w | 60 | 93,8% |  | 56 | 88,9% |  |
|  | >=1x/d | 3 | 4,7% |  | 0 | 0,0% |  |
| **Salad and raw vegetables** | <1x/w | 3 | 4,6% |  | 4 | 6,3% | 0.094 |
|  | 1-6x/w | 41 | 63,1% |  | 49 | 77,8% |  |
|  | >=1x/d | 21 | 32,3% |  | 10 | 15,9% |  |
| **Cooked vegetables** | <1x/w | 8 | 13,1% |  | 16 | 25,4% | 0.096 |
|  | 1-6x/w | 47 | 77,0% |  | 45 | 71,4% |  |
|  | >=1x/d | 6 | 9,8% |  | 2 | 3,2% |  |
| **Fruits** | <1x/w | 0 | 0,0% |  | 12 | 19,0% | <0.001 |
|  | 1-6x/w | 19 | 30,2% |  | 35 | 55,6% |  |
|  | >=1x/d | 44 | 69,8% |  | 16 | 25,4% |  |
| **Fast food** | <1x/w | 63 | 98,4% |  | 56 | 91,8% | 0.083 |
|  | 1-6x/w | 1 | 1,6% |  | 5 | 8,2% |  |
|  | >=1x/d | 0 | 0,0% |  | 0 | 0,0% |  |
| **Ready Meal** | <1x/w | 61 | 98,4% |  | 48 | 82,8% | 0.003 |
|  | 1-6x/w | 1 | 1,6% |  | 10 | 17,2% |  |
|  | >=1x/d | 0 | 0,0% |  | 0 | 0,0% |  |
| **Whole wheat bread** | <1x/w | 13 | 20,6% |  | 31 | 50,8% | <0.001 |
|  | 1-6x/w | 39 | 61,9% |  | 28 | 45,9% |  |
|  | >=1x/d | 11 | 17,5% |  | 2 | 3,3% |  |
| **Oatmeal, muesli** | <1x/w | 35 | 57,4% |  | 54 | 88,5% | <0.001 |
|  | 1-6x/w | 17 | 27,9% |  | 7 | 11,5% |  |
|  | >=1x/d | 9 | 14,8% |  | 0 | 0,0% |  |
| **Curd, yoghurt** | <1x/w | 9 | 15,0% |  | 30 | 49,2% | <0.001 |
|  | 1-6x/w | 35 | 58,3% |  | 27 | 44,3% |  |
|  | >=1x/d | 16 | 26,7% |  | 4 | 6,6% |  |
| **Milk** | <1x/w | 12 | 19,4% |  | 26 | 42,6% | 0.006 |
|  | 1-6x/w | 20 | 32,3% |  | 20 | 32,8% |  |
|  | >=1x/d | 30 | 48,4% |  | 15 | 24,6% |  |
| **Cheese** | <1x/w | 3 | 4,7% |  | 9 | 14,3% | 0.030 |
|  | 1-6x/w | 49 | 76,6% |  | 50 | 79,4% |  |
|  | >=1x/d | 12 | 18,8% |  | 4 | 6,3% |  |
| **Chips, pretzel sticks** | <1x/w | 57 | 89,1% |  | 46 | 75,4% | 0.086 |
|  | 1-6x/w | 7 | 10,9% |  | 13 | 21,3% |  |
|  | >=1x/d | 0 | 0,0% |  | 2 | 3,3% |  |
| **Softdrinks** | <1x/w | 58 | 92,1% |  | 30 | 49,2% | <0.001 |
|  | 1-6x/w | 3 | 4,8% |  | 16 | 26,2% |  |
|  | >=1x/d | 2 | 3,2% |  | 15 | 24,6% |  |
| **Water** | <1x/w | 3 | 4,9% |  | 14 | 23,0% | <0.001 |
|  | 1-6x/w | 1 | 1,6% |  | 13 | 21,3% |  |
|  | >=1x/d | 57 | 93,4% |  | 34 | 55,7% |  |
| **Beer** | <1x/w | 39 | 60,0% |  | 25 | 40,3% | 0.009 |
|  | 1-6x/w | 22 | 33,8% |  | 22 | 35,5% |  |
|  | >=1x/d | 4 | 6,2% |  | 15 | 24,2% |  |
| **Sugar for tea and coffee** | <1x/w | 52 | 80,0% |  | 23 | 37,7% | <0.001 |
|  | 1-6x/w | 2 | 3,1% |  | 6 | 9,8% |  |
|  | >=1x/d | 11 | 16,9% |  | 32 | 52,5% |  |
